# Editorial: Plant products for antiviral therapeutics

**DOI:** 10.3389/fphar.2022.1039183

**Published:** 2022-10-17

**Authors:** Anirban Mandal, Banasri Hazra, Vijay Kumar Prajapati, Paul F. Moundipa

**Affiliations:** ^1^ Department of Microbiology, Mrinalini Datta Mahavidyapith, Kolkata, India; ^2^ Department of Pharmaceutical Technology, Jadavpur University, Kolkata, India; ^3^ Department of Biochemistry, School of Life Sciences, Central University of Rajasthan, Ajmer, India; ^4^ Laboratory of Pharmacology and Toxicology, Faculty of Science, AEFAS, University of Yaoundé I, Yaounde, Cameroon

**Keywords:** viral infection, antiviral drug, plant-based antiviral therapeutics, antiviral plants, coronavirus

Diseases caused by microbial infections continue to pose major public health challenges despite the enormous modernization of therapeutic strategies to alleviate human health disorders. From time to time, numerous such afflictions due to HIV, hepatitis B & C, herpes simplex, dengue, Ebola, and influenza viruses raised universal concern, underscored by the recent COVID-19 pandemic caused by SARS-CoV-2 (Mandal et al., 2021). Although some antiviral drugs or cocktails of drugs are available to combat these viruses, nevertheless, most of these medications showed side effects and other drawbacks like low antiviral activity, genotype-dependent efficacy, and the emergence of drug-resistant mutants. Therefore, the development of more suitable therapeutics in this field is certainly a need of the hour. Historically, medicinal herbs and natural products are the mainstay of general healthcare systems around the globe and are considered an integral part of the cultural heritage of mankind. Truly, the plant kingdom is an invaluable repository of countless phytochemicals with distinctive molecular frameworks, which provide clues on the core structures as templates for the development of novel drug molecules. Thus, quite a number of such traditional medicinal plants yielded a variety of drugs enlisted in the modern pharmacopeia. In fact, a substantial number of FDA-approved drugs are based on natural products or their derivatives (Atanasov et al., 2021).

Time-honoured herbal prescriptions practised worldwide, such as Traditional Chinese Medicines (TCM), Indian Ayurvedic and Middle-eastern Unani systems, and various regional folkloric treatments in Africa and elsewhere continue to fulfil basic healthcare requirements in developing countries (Mandal et al., 2020). As for the Western people, a resurgence in popularity of natural products for health promotion have been observed in the past few decades (World Health Organization, 2019). In fact, many medicinal plant extracts are now prescribed for the treatment of chronic ailments. Therefore, a strategic approach to combat viral infections would be to explore the empirical records of medicinal practices enshrined in worldwide cultural traditions and develop this knowledge with necessary support from technological advancement achieved in the modern era. Under this context, the present volume entitled *Plant products for antiviral therapeutics* was published, comprising eight Original Research articles and seven Reviews/Mini-reviews, which will be briefly introduced in this editorial.

TCM is one of the world’s classical therapeutic modalities, gaining substantial popularity worldwide. Actually, TCM is known to utilize the characteristics of its multiple components and produces a cocktail-like effect through integrated regulation of multiple biochemical targets to block viral infections (Tang et al., 2018). Ping et al. explored the efficacy of Jing Guan Fang (JGF) to prevent SARS-CoV-2 infection. The authors showed that JGF, a TCM formula comprising five commonly used herbs, effectively blocked syncytium formation and inhibited SARS-CoV-2 plaque formation. They demonstrated the underlying mechanism of JGF working *via* induction of lysosome-dependent ACE2 degradation and inhibition of TMPRSS2 expression in the lung tissue of mice. Additionally, a clinical study conducted on human volunteers recommended that JGF would be a useful preventative measure for frontline medical staff or people who have had high-risk exposure to COVID-19 cases.

Women of childbearing age are widely susceptible to common fungal infections as well as Herpes simplex virus type 2 (HSV-2). Since many years, a TCM called JieZe-1 had been used for the treatment of female lower reproductive tract infections. Hence, Duan et al. had explored a prescription of JieZe-1 composed of authenticated botanical ingredients as specified in ancient Chinese scripture and reported its preventive and therapeutic efficacy on HSV-2 patients in Chinese hospitals (Duan et al., 2020). Presently, Duan et al. could further validate the anti-HSV-2 effect of JieZe-1 in experiments conducted *in vitro*. Further, they optimized a mouse model of genital herpes to clarify the underlying mechanism of its anti-HSV-2 effect and found that JieZe-1 inhibited membrane fusion and TLR signaling pathways *in vivo*. Thus, the study by Duan et al. was an experimental corroboration of the anti-HSV-2 effect of JieZe-1. Finally, they suggested that the antiviral effect could be exerted through the synergic interaction of its herbal components, reflecting the scientific implication of TCM.

For using a complex mixture of natural products, it is crucial to identify the target proteins of TCM and evaluate the mechanism of action. Lai et al. explored a standardized composition of Ganghuo Kanggan Decoction (GHKGD) and its components for prospective activity against the influenza virus, using a series of network pharmacology involving compound screening, target prediction, and pathway enrichment analysis. Hence, a compound-target-disease network was constructed and analyzed to explore the relevant mechanism. Thus, they could identify 116 active compounds and 17 potential therapeutic targets of GHKGD. Further, they established a mouse model to study the therapeutic efficacy of this TCM against viral pneumonia caused by the H1N1 influenza virus. Thus, GHKGD treatment was found to balance the pro- and anti-inflammatory cytokine levels, thereby alleviating mouse lung inflammation due to influenza virus infection. Furthermore, they performed *in vivo* experiments to reveal the synergistic action of multi-component and multi-target characteristics of GHKGD and offered a theoretical basis for prospective drug development against this contagious respiratory viral disease.

Viral pneumonia is one of the most pernicious respiratory ailments; TCM treatments are documented to manage infected patients. The multi-target action of TCMs could be elucidated by way of proteomics (Lao et al., 2014). Hence, Wei et al. utilized this approach to precisely clarify the efficacy of Hou Yan Qing (HYQ) oral liquid, subsequent to its validation in a mouse model of pneumonia caused by influenza A virus (H1N1), *in vivo*. Experimental results indicated that HYQ treatment would cause elevation of galectin-3-binding protein and glutathione peroxidase 3 to produce distinctive effects on viral pneumonia patients. This TCM composition of four authentic plant samples as per Chinese Pharmacopoeia 2020, showed multiple effects on the complement system and inflammatory processes; hence, it could be a promising strategy for personalized management of viral pneumonia.

Indian Ayurveda is a well-documented traditional system of complementary herbal medicines recommended for chronic ailments and general wellness improvement (Sen and Chakraborty, 2015). Since SARS-CoV-2 infection targets multiple organs, a holistic approach may be an answer to the current crisis, as prescribed in Ayurveda. Balkrishna et al. explored a prospective Ayurvedic medicine called Giloy Ghanvati (GG), an oral tablet prepared from *Tinospora cordifolia* (Willd.) Hook. f. & Thomson, a well-known tropical plant widely used against various diseases in the Indian subcontinent. GG is a time-tested formulation recommended as a natural immunity booster to improve overall health, particularly to inhibit SARS-CoV-2 infection during the Covid-19 pandemic. Balkrishna et al. developed the SARS-CoV-2 spike-protein induced disease phenotype in a humanized zebrafish model. They studied several parameters to prove that the disease symptoms due to SARS-CoV-2 spike protein expression were substantially ameliorated by treatment of the afflicted fishes with aqueous extract of GG. Also, HPLC analysis of GG extract could detect the major phytoconstituents as cordifolioside A, magnoflorine, β-ecdysone, and palmatine, already known for anti-inflammatory and antiviral activities. Thus, the findings correlated well with the potential role of these constituents of GG against SARS-CoV-2 viral symptoms.

*Vigna radiata* (L.) R. Wilczek (mung bean legume), a common functional food in Oriental culture, is sparsely studied for its antiviral potential. Using appropriate techniques, Lo et al. prepared *Vigna radiata* extract (VRE) from seed coats of mung beans and investigated the antiviral activity and mechanism of action of VRE against the influenza virus. They found that VRE mainly targets viral entry and release by interfering with hemagglutinin and neuraminidase activity and significantly inhibited viral replication in a concentration-dependent manner. The authors concluded that VRE is a multi-step inhibitor for both mammalian H1N1 and avian H6N1 subtypes of influenza virus and demonstrated its broad-spectrum potential as a preventive and therapeutic agent against influenza virus.

*Buddleja indica* Lam. is an evergreen shrub native to Madagascar, locally known as a topical antiseptic. The investigation by Youssef et al. is a rare study to establish the traditional medicinal claims on *Buddleja spp.* of plants, probably owing to its richness of phenolic acids and flavonoids with antioxidant and hepatoprotective activity. Youssef et al. prepared *Buddleja indica* Lam. leaf extract with credible anti-tubercular and anti- *Helicobacter pylori* activity and isolated caffeic acid, quercetin 7-O-β-D-glucoside and kaempferol. Further, *in silico* study on molecular docking of these compounds showed the highest fitting score in proteins implicated for bacterial infection and the occurrence and progression of SARS-CoV-2 virus.

Saha et al. used *in silico* tools to identify RNA dependent RNA polymerase (RdRp) inhibitors as prospective drug candidates against COVID-19. A collection of 248 plant-derived molecules with strong antiviral activity were subjected to molecular docking against the catalytic sub-unit of RdRp. Pharmacokinetics analysis and molecular dynamics simulation of the best-docked compounds showed that tellimagrandin I, saikosaponinB2, hesperidin & (-)-epigallocatechin gallate were the most prominent ones with a strong binding affinity towards RdRp. Overall, the study unveiled saikosaponin B2 to serve as a prospective molecule for the development of effective therapy against COVID-19, as it was one of the top inhibitors of RdRp, the crucial replication protein. Such an approach combined with biological testing can substantially speed up the drug discovery process from plant products.

Huang et al. reviewed an updated selection of phytochemicals active against MERS-CoV, SARS-CoV, or SARS-CoV-2, along with their specific molecular targets and mechanisms of action. Omrani et al. focussed on phylogenetic and taxonomic similarities of viruses, like influenza, MERS-CoV, SARS-CoV, and SARS-CoV-2, and pertinent mechanisms of action. Accordingly, they presented 130 plant-derived compounds with therapeutic potential to combat the currently relevant infections caused by these respiratory viruses. Alam et al. reviewed prospective anti-COVID plant products undergoing clinical trials to address a pandemic-like situation happening in the future. Anywar et al. enlisted medicinal plant species indigenous to Africa and Asia as potential antiviral agents, especially for HIV and SARS-CoV-2. Abubakar et al. reviewed selected medicinal plants and compounds implicated in the mitigation of viral invasion either *via* direct or indirect modulation of ACE2 activity to ameliorate COVID-19. The review article by Hu et al. focused on herbal products with reported therapeutic efficacy against experimental models of influenza, respiratory syncytial virus, and coronavirus - the three types of viruses eliciting pathologic manifestations of viral pneumonia. Severe respiratory distress and associated lung problems due to SARS-CoV-2 infection often create cardiovascular complications, leading to severe myocardial injuries. Hence, Beura et al. dealt with the significance of inhibiting platelet-mediated thrombus formation and discussed the role of anti-platelet and anti-thrombotic phytochemicals available from medicinal plants.

Taken together, it remains to carry on further research and clinical trials on plenty of plant products to get novel therapeutics suitable for clinical application against viral diseases.
